# Risk factors of primary postpartum hemorrhage among postnatal mothers in the public hospital of southern Tigray, Ethiopia, 2019: A case-control study

**DOI:** 10.3389/fgwh.2023.1039749

**Published:** 2023-02-14

**Authors:** Getachew Muluye, Abeba Gashaw, Lebasie Woretaw, Biniam Girma, Tarekegn Tumebo

**Affiliations:** ^1^ Department of Midwifery, College of Health Sciences, Mekelle University, Mekelle, Ethiopia; ^2^ Department of Maternal and Child Health, Amdework Primary Hospital, Amdework, Ethiopia; ^3^ Department of Environmental Health, College of Medicine and Health Sciences, Wollo University, Dessie, Ethiopia; ^4^ Department of Midwifery, College of Medicine and Health Sciences, Wachemo University, Hosaena, Ethiopia

**Keywords:** risk factors, primary postpartum hemorrhage, postnatal, case-control, Ethiopia

## Abstract

**Background:**

Postpartum hemorrhage is the most common direct cause of maternal mortality and morbidity; among them, primary postpartum hemorrhages are an enormous element. Although it has an incredible impact on maternal lifestyle, this is the most neglected area in Ethiopia and there is a lack of studies achieved within the study area. So this study aimed to identify the risk factors of primary postpartum hemorrhage among postnatal mothers in public hospitals in southern Tigray, Ethiopia, 2019.

**Methods:**

Institution-based unmatched case-control study design was conducted on 318 (106 case and 212 controls) postnatal mothers in public hospitals of Southern Tigray from January to October 2019. We used a pretested, structured interviewer-administered questionnaire and a chart review to collect the data. Bivariate and multivariable logistic regression models were used to identify the risk factors. *P*-value ≤ 0.05 was considered statically significant for both steps and an odds ratio with a 95% confidence level was used to see the strength of association.

**Results:**

Abnormal third stage of labor [adjusted odds ratio = 5.86, 95% confidence interval (2.55–13.43), *P* = 000], cesarean section [adjusted odds ratio = 5.61, 95% confidence interval (2.79–11.30), *P* = 0.000], lack of active management of third-stage labor [adjusted odds ratio = 3.88; 95% confidence interval (1.29–11.60), *P* = 0.015], absence of labor monitoring by partograph [adjusted odds ratio = 3.82, 95% confidence interval (1.31–11.09), *P* = 0.014], lack of antenatal care [adjusted odds ratio = 2.76, 95% confidence interval (1.13–6.75), *P* = 0.026] and complications during pregnancy [adjusted odds ratio = 2.79, 95% confidence interval (1.34–5.83), *P* = 0.006] were found to be risk factors for primary postpartum hemorrhage.

**Conclusion:**

In this study complications and lack of maternal health interventions in the course of the antepartum and the intrapartum period were found to be risk factors for primary postpartum hemorrhage. A strategy for improving essential maternal health services and detecting and handling complications in a timely manner will help to prevent primary postpartum hemorrhage.

## 1. Introduction

Primary postpartum hemorrhage (PPH) is defined by the American College of Obstetricians and Gynecologists (ACOG) as blood loss of 1,000 ml or more, or blood loss that was accompanied by signs or symptoms of hypovolemia occurring within 24 h after birth, regardless of the mode of delivery (1). Defining PPH as the amount of blood loss is problematic, since the amount of blood loss is much less important than the effect it has on the woman and underestimation of blood loss, and some suggest that a clinically applicable definition should be considered (2, 3).

Postpartum hemorrhage is a major cause of maternal mortality and accounts for one-quarter of all maternal deaths in developing countries (4–6), and serious morbidities such as severe anemia, complications of multiple blood transfusions, loss of fertility secondary to peripartum hysterectomy and a psychological sequel and poor growth and development of their children (7–9).

Even though primary PPH occurs in all geographic regions, the majority of maternal deaths because of PPH take place in developing countries (10) and this disparity has been attributed to differences in access to reproductive health services, the availability of skilled personnel attending deliveries, appropriate equipment and supplies and the timely receipt of needed interventions whilst obstetric emergencies arise (11, 12). Ethiopia's maternal mortality ratio is high at the national level (13), with 412 deaths per 100,000 live births in the 2016 Ethiopian Demographic Health Survey (EDHS) (14) of which 25%–30% are attributed to PPH (15).

Reducing maternal mortality is one of the hot agendas globally and nationally. By 2030, reduce the global maternal mortality ratio to less than 70 per 100,000 live births, and no country should have a maternal mortality rate of more than twice the global average (16). So, identifying the gap and improving health care during pregnancy, childbirth and postpartum is an essential step toward achieving this goal (17). PPH remains the leading cause of maternal mortality in Ethiopia, despite a significant improvement in maternal health care services to address concerns related to pregnancy and childbirth (18).

A review of the works of the literature revealed that maternal age and educational level (9, 19, 20), occupations and residence (20), partnerships with male partners, and a husband's educational level (21) were identified as risk factors for primary PPH. The likelihood of PPH increased among women with a history of PPH (18, 22, 23) pregnancy-induced hypertension (PIH) (22, 24, 25), pre-existing anemia (18, 22), Antepartum hemorrhage (APH) (26), multiple pregnancies and macrosomic baby, polyhydramnios or one with multiple fibroids (22, 27–29). Besides, lack of antenatal care (ANC) throughout pregnancy and inadequate birth interval (7, 19, 30), child delivery at home or on the way and lack of labor monitoring by partograph (15), duration of labor (31, 32), a protracted third stage of labor (33–35), emergency or referral admission (7) were discovered to be a risk factor for primary PPH. Moreover, repeated use of obstetric interventions like induced abortion, labor induction, and augmentation, instrumental delivery, repeated cesarean section (CS), and episiotomy may be responsible for the increasing incidence of postpartum hemorrhage (29, 36, 37).

However, active management of the third stage of labor (AMTSL) has been demonstrated to reduce the risk of PPH (19, 38).

From this, we can infer that there are modifiable risk factors and these risk factors must first be identified, and then appropriate and specific methods must be developed to enable the prevention, diagnosis, and management of primary PPH.

Although it has a substantial impact on maternal life, this is the most neglected area, and health care practitioners and the community in Ethiopia have paid little attention and effort to PPH. As a result, this study will be useful as a contribution to better attention and efforts by all concerned sectors as well as for evaluating the current system and future planning and intervention for effective policy and health program decisions to prevent maternal morbidity and mortality linked to PPH.

**Figure 1:**
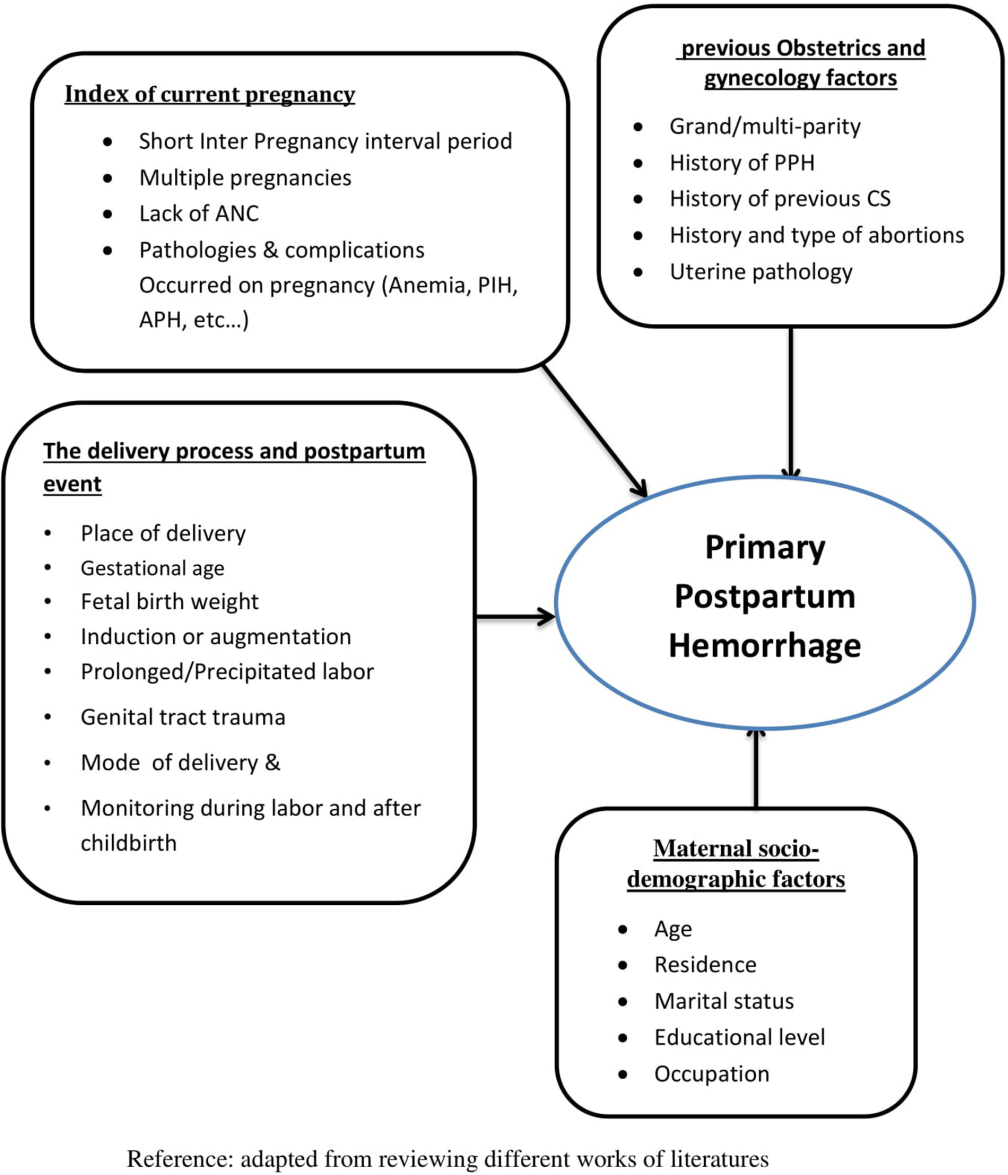
Conceptual framework on risk factors of primary postpartum hemorrhage.

A conceptual framework showed an expected relationship between variables (Figure 1).

## 2. Materials and methods

### 2.1. Study area and period

A hospital-based unmatched case-control study design was conducted in public hospitals in Southern Tigray from January to October 2019. Southern Zone is one of the seven zones in the region of Tigray regional State. The southern zone is bordered on the south and west by the Amhara region, on the north by the southeastern zone, and on the east by the Afar region and it is located around 172 km south of Mekelle city and 600 km north of Addis-Ababa capital city of Ethiopia. Based on the 2012 census conducted by the central statics agency of Ethiopia, this zone has a total population of 1,138,440 of whom 576,352 are females. The zone has two general hospitals (Lemlem Karl general hospital and Alemata general hospital) and three primary hospitals such as Korem primary hospital, Mekoni primary hospital, and Adishu primary hospital, and 34 health centers and 65 health posts. Maternity services, along with other services, are available in all hospitals.

### 2.2. Populations

#### 2.2.1. Source population

All postnatal mothers who were admitted to the postnatal ward of public hospitals in Southern Tigray.

#### 2.2.2. Study population

All postnatal mothers who were admitted to the postnatal ward of the public hospitals at Southern Tigray during the time of data collection.

### 2.3. Ascertainment of cases and controls

♣ Cases: were all postnatal mothers diagnosed as having primary PPH by attending health care providers and admitted to the postnatal ward during the study period
♣ Controls: were postnatal mothers without primary PPH confirmed by attending health care providers and admitted to the postnatal ward during the study period.

### 2.4. Sample size determination and sampling technique

#### 2.4.1. Sample size determination

The desired sample size for the study was determined using EPI INFO version 7.1 statistical software using the double population proportion formula for unmatched case-control by taking a history of PPH as a risk factor from the previous study (22), where the proportion of exposure among cases was 8.3% with odds ratio 8.97, with the assumptions of a 95% confidence interval (CI), an 80% power, and a 2:1 control to case ratio. Finally, it gave us a sample size of 318 (212 controls and 106 cases).

#### 2.4.2. Sampling technique and procedure

**Figure 2:**
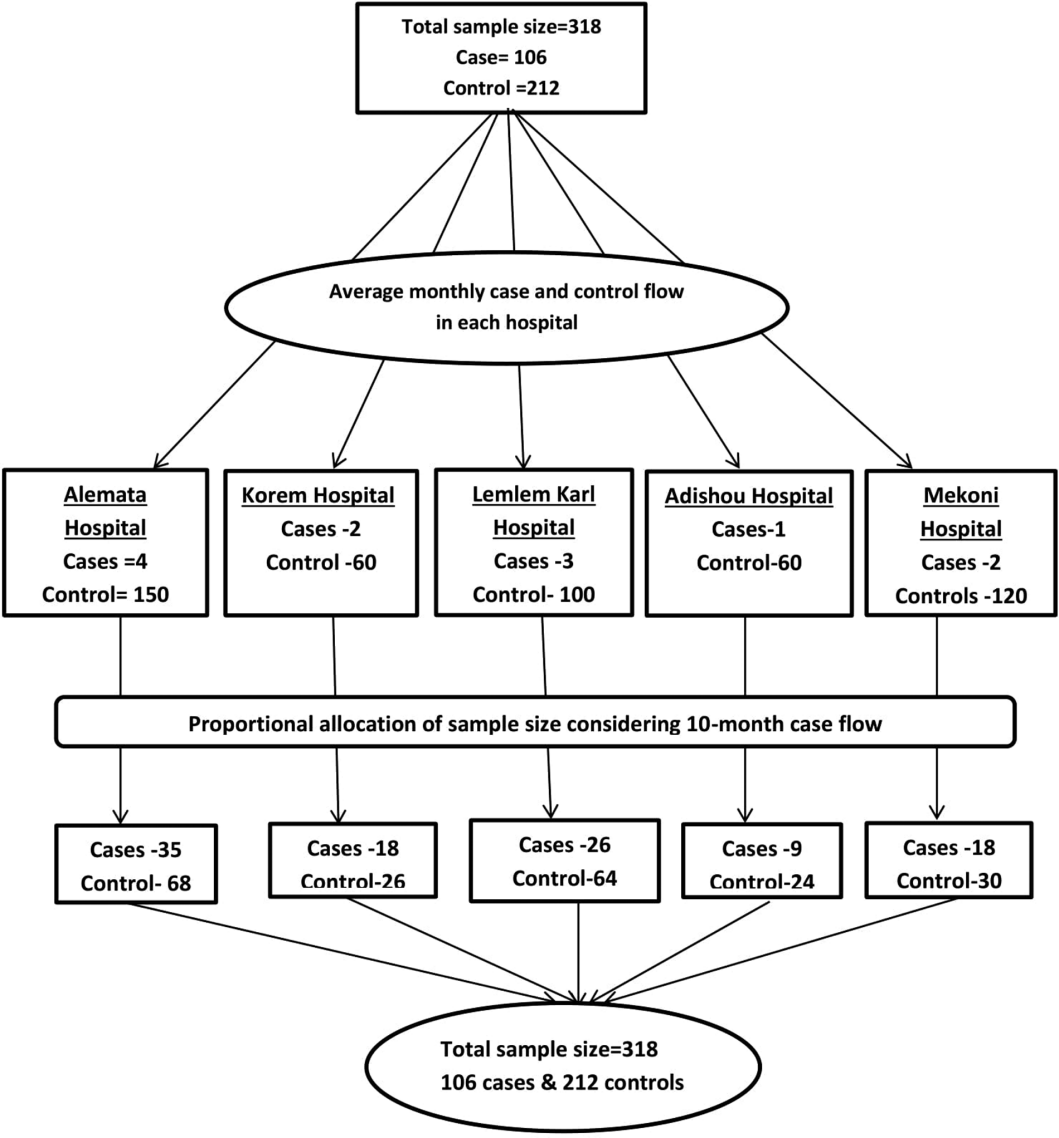
Schematic representation of the sampling technique.

All five public hospitals in the Zone were included in the study. According to a case flow during 10 months, the study hospitals received a proportionate share of the sample size (Cases and controls). Women with primary PPH who meet the inclusion criteria were recruited consequently as cases until the calculated sample size was attained; for every case, two women without primary PPH and in their 24-h postpartum period who meet the inclusion criteria were chosen as controls during the study period (Figure 2).

### 2.5. Data collection tools and procedures

The data were collected through face-to-face interviews and a review of the client's medical record using pretested structured questionnaires and a checklist. We developed the questionnaire after reviewing different literature and it included information related to the maternal socio-demographic condition, obstetric and gynecologic history, current pregnancy characteristics, and intrapartum and postpartum events. Seven BSc degree holder midwives who were working in another health facility collected data from January to October 2019. Two BSc health officer supervisors and a principal investigator (PI) are in charge of overseeing the data collection procedure and ensuring the questionnaire for its completeness and consistency.

### 2.6. Data quality control and assurance

The questionnaire was created in English and translated into Tigrigna which is the local language of the study area. Then it was translated back into English by language experts to check for consistency. The questionnaire was reviewed by a senior researcher and the Pre-test was conducted ahead of the actual data collection at 10% of the sample size at a nearby hospital (Quiha General Hospital) which is not included in the study to see the appropriateness of the tool and feedbacks were incorporated accordingly. A 3-day training was given to the data collector and supervisor on general information on the aim of the study, techniques of sampling, and data collection methods. The PI and supervisors had site supervision. The data was checked before and after the data entry stage to verify its completeness and double data entry was done.

### 2.7. Data processing and analysis

The collected data were entered *via* Epi-Data version 4.6.0.0 and exported to SPSS version 23 software for analysis. We employed descriptive statistics to organize, summarize and present the results using frequencies, percentages, or in the form of texts. Bivariate and multivariate logistic regression models were used to identify the risk factors for the primary PPH. Variables having a *P*-value of less than 0.05 is considered significant variables for both steps. An adjusted odd ratio (AOR) with a 95% CI was considered to measure the strength of association between the outcome and independent variables. Hosmer Lemeshow tests were conducted to check model goodness of fit.

### 2.8. Study variables

**Dependent variables**: Primary postpartum hemorrhage.

**Independent variables include**: (1) Socio-demographic characteristics: Age, ethnicity, marital status, residence, educational level, occupation, and husband's educational level. (2) Gynecological and obstetrical history: History of PPH, history of cesarean delivery, uterine pathology, history and type of abortions. (3) Characteristics of current pregnancy: Gravidity, parity, inter-pregnancy interval, ANC, multiple gestations, and any complications that occurred during pregnancy such as anemia, PIH and APH. (4) Intrapartum and postpartum events: Way of admission, place of delivery, induction or augmentation, duration of labor, newborn birth weight, genital tract trauma, mode of delivery and monitoring during labor with partograph and after childbirth like safe childbirth checklist.

### 2.9. Operational definition

**Antenatal care (ANC)**: is the care provided by skilled healthcare professionals to women throughout their pregnancy (39). Mothers are said to have ANC if they visited health institutions at least once during the pregnancy period.

**AMTSL**: A prophylactic lifesaving intervention that is the administration of a uterotonics agent immediately after the birth of the baby (40).

**The prolonged third stage of labor**: if not completed within 30 min of the birth (28).

**Labor not monitored with partograph**: if the three components of partograph were not filled out correctly or not documented to maternal chart at all (42).

**Anemia**: A hemoglobin (HGB) level below 11 g/dl (43).

### 2.10. Ethics approval and informed consent

All the methods carried out in this study were approved by Mekelle University Health Research Ethics Review Committee (HRERC) (Reference number: ERC 1381/2019) and certifies that the study was performed in accordance with the ethical standards. Ethical clearance was obtained from the institutional review board (IRB) of Mekelle University. An official letter obtained from the Tigray regional health bureau was submitted to each hospital and data was collected after permission was obtained from the medical director of the hospital. The purpose of the study and the right of the respondent to withdraw and not participate or to stop participation at any time was informed to the participant. The informed consent of the participant in the study was secured before conducting the interview. Participants took part voluntarily. Anonymity and confidentiality were maintained by excluding personal identifiers from the data collection tool and records were kept strictly confidential.

## 3. Results

### 3.1. Socio-demographic characteristics of the study participants

A total of 318 mothers (106 cases and 212 controls) were interviewed for this study and yielding a response rate of 100%. The mean ages for cases and controls were 29.77 years (SD 6.96 years) and 26.4 years (SD 5.8 years), respectively. There was no statically significant difference between cases and controls in the percentage of older age mothers (age ≥ 35), which was 17.9% vs. 12.7% respectively. Moreover, more than half (54.7%) of respondents had attained either a low level or no schooling (Table 1).

**Table 1:** Socio-demographic characteristics of study participants with and without primary postpartum hemorrhage in the public hospital of southern Tigray, Ethiopia, 2019.

Variables	Categories	Cases *n* (%)	Controls *n* (%)	Total *N* = 318	*P*-value
Age group (year)	20–34	79 (74.5)	173 (81.6)	252 (79.2)	0.339
	<20	8 (7.5)	12 (5.7)	20 (6.3)	
	≥35	19 (17.9)	27 (12.7)	46 (14.5)	
Residence	Urban	35 (33.0)	84 (39.6)	119 (37.4)	0.251
	Rural	71 (67.0)	128 (60.4)	199 (62.6)	
Ethnicity	Tigray	89 (84.0)	175 (82.5)	264 (83.0)	0.777
	Amhara	12 (11.3)	29 (13.7)	41 (12.9)	
	Afar	5 (4.8)	8 (3.7)	13 (4.1)	
Religion	Orthodox	88 (83.0)	166 (78.3)	254 (79.9)	0.074
	Muslim	18 (17)	46 (21.7)	64 (20.2)	
Marital status	Currently in relationship	103 (97.2)	204 (96.2)	307 (96.5)	0.664
	Not in relationship	3 (2.8)	8 (3.8)	11 (3.5)	
Educational level	No education	37 (34.9)	53 (25.0)	90 (28.3)	0.241
	Primary	25 (23.6)	59 (27.8)	84 (26.4)	
	Secondary	15 (14.2)	42 (19.8)	57 (17.9)	
	College and above	29 (27.4)	58 (27.4)	87 (27.4)	
Occupation	Employed	21 (19.8)	62 (29.2)	83 (26.1)	0.105
	Unemployed	85 (80.2)	150 (70.8)	235 (73.9)	
Husband's educational level (*N* = 307)	No education	26 (25.2)	39 (19.1)	65 (21.2)	0.331
	Primary	20 (19.4)	45 (22.1)	65 (21.20)	
	Secondary	22 (21.4)	34 (16.7)	56 (18.2)	
	College and above	35 (34.00)	86 (42.2)	121 (39.4)	

Maximum maternal age = 41 and minimum maternal age = 17 years; not in a relationship = unmarried, divorced, and widowed; n, number; %, percent.

### 3.2. Past obstetrics and gynecology history of the study participants

Two third (67.0%) of the study participants were multiparous (had given birth 2–4 times) which was a comparable proportion among cases and controls (66.0% vs. 67.5%) respectively. About 12.9% of cases and 8.3% of controls had a previous history of cesarean delivery with no statically significant difference (*P* = 0.23) (Table 2).

**Table 2:** Obstetrics and gynecologic history of study participants with and without primary postpartum hemorrhage in the public hospital of southern Tigray, Ethiopia, 2019.

Variable	Category	Cases *n* (%)	Controls *n* (%)	Total *N* = 318	*P*-value
Parity	Primipara	21 (19.8)	53 (25.0)	74 (23.3)	0.135
	Multipara	70 (66.0)	143 (67.5)	213 (67.0)	
	Grand multipara	15 (14.2)	16 (7.5)	31 (9.7)	
History of abortion	Yes	25 (23.6)	43 (20.3)	68 (21.4)	0.435
	No	81 (76.4)	169 (79.7)	250 (78.6)	
Type of abortion (*n* = 68)	Spontaneous	19 (76.0)	35 (81.4)	54 (79.4)	0.596
	Induced	6 (24.0)	8 (18.6)	14 (20.6)	
History of Cesarean delivery (*n* = 244)	Yes	11 (12.9)	13 (8.2)	24 (9.8)	0.234
	No	74 (87.1)	146 (91.8)	220 (90.2)	
History of PPH (*n* = 244)	Yes	7 (8.2)	8 (5.0)	15 (6.1)	0.231
	No	78 (91.8)	151 (95.0)	229 (93.9)	
Uterine abnormality	Yes	2 (1.9)	4 (1.9)	6 (1.9)	0.986
	No	104 (98.1)	208 (98.1)	312 (98.1)	

N, number; %, percentage; Grand multipara: had given birth ≥5 times.

### 3.3. Current pregnancy characteristics of the study participants

The vast majority of pregnancies were singletons and the proportions of cases and controls were comparable (98.0% vs. 97.6%) respectively. In contrast, about 89.6% of study participants had ANC follow-up, and the cases had a lower proportion (81.1%) when compared to controls (93.9%). The proportions of complications during the current pregnancy among cases were nearly twice as high as the proportion among controls (24.5% vs. 13.7%) (Table 3).

**Table 3:** Index pregnancy characteristics of study participants with and without primary postpartum hemorrhage in the public hospital of southern Tigray, Ethiopia, 2019.

Variable	Categories	Cases *n* (%)	Controls *n* (%)	Total *N* (%)	*P*-value
Inter-pregnancy interval (months) (*n* = 244)	<24	29 (34.1)	45 (28.3)	74 (30.3)	0.346
	≥24	56 (65.9)	114 (71.7)	170 (69.7)	
Type of pregnancy	Singleton	104 (98.1)	207 (97.6)	311 (97.8)	0.787
	Multiple	2 (1.9)	5 (2.4)	7 (2.2)	
ANC visit	Yes	86 (81.1)	199 (93.9)	285 (89.6)	0.000
	No	20 (18.9)	13 (6.1)	33 (10.4)	
Total number of ANC visits	<4	44 (51.2)	84 (42.2)	128 (44.9)	0.167
	≥4	42 (48.8)	115 (57.8)	157 (55.1)	
Complications during pregnancy (*n* = 318)	Yes	26 (24.5)	29 (13.7)	55 (17.3)	0.016
	No	80 (75.5)	183 (86.3)	263 (82.7)	
Pregnancy Induced Hypertension (PIH) (55)	Yes	10 (38.5)	7 (24.1)	17 (30.9)	0.251
	No	16 (61.5)	22 (75.9)	38 (69.1)	
Anemia (*n* = 55)	Yes	6 (23.1)	10 (34.5)	16 (29.1)	0.352
	No	20 (76.9)	19 (65.5)	39 (70.9)	
Antepartum Hemorrhage (APH) (*n* = 55)	Yes	8 (30.8)	5 (17.2)	13 (23.6)	0.238
	No	18 (69.2)	24 (82.8)	42 (76.4)	
Others* (*n* = 55)	Yes	2 (3.6)	7 (12.7)	9 (16.4)	0.235
	No	24 (96.4)	22 (87.3)	46 (83.6)	

Others*: polyhydraminous = 3; Congenital anomaly = 4; Hyperemesis gravidarum = 2. Inter-pregnancy interval (IPI) (short IPI < 24-month duration).

### 3.4. Intrapartum and postpartum characteristics of the study participants

About 95.3% were institutional delivery, and the remaining were either home or on the way to the institution. The proportion of home delivery among cases was higher when compared to controls (8.5% vs. 2.8%). Regardless of the category, the majority of study participants gave birth to infants weighing between 2,500 and 3,999 g. Abnormality in the 3rd stage of labor was present in about 23.6% of cases and 6.6% of controls with a significant difference between the group (*P* = 0.000) (Table 4).

**Table 4:** Intrapartum and postpartum characteristics of study participants with and without primary postpartum hemorrhage in the public hospital of southern Tigray, Ethiopia, 2019.

Variable	Categories	Cases *n* (%)	Controls *n* (%)	Total *N* = 318	*P*-value
Place of birth	Institutional	97 (91.5)	206 (97.2)	303 (95.3)	0.025
	Non-institutional	9 (8.5)	6 (2.8)	15 (4.7)	
Type of admission	Non-referral	84 (79.2)	186 (87.7)	270 (84.9)	0.046
	Referral	22 (20.8	26 (12.3)	48 (15.1)	
Way of labor initiated	Spontaneously	91 (85.8)	197 (92.9)	288 (90.6)	0.042
	Induced	15 (14.2)	15 (7.1)	30 (9.4)	
Gestational age (weeks) (*N* = 313)	Term	88 (84.6)	187 (89.5)	275 (87.9)	0.378
	Preterm	11 (10.6)	13 (6.2)	24 (7.7)	
	Post-term	5 (4.8)	9 (4.3)	14 (4.5)	
Mode of delivery	SVD	67 (63.2)	178 (84.0)	245 (77.0)	0.000
	Cesarean delivery	34 (32.1)	25 (11.8)	59 (18.6)	
	Assisted vaginal delivery	5 (4.7)	9 (4.2)	14 (4.4)	
FBW (grams) (*N* = 316)	2,500–3,999	77 (74.0)	165 (77.8)	242 (76.6)	0.608
	<2,500	14 (13.5)	28 (13.2)	42 (13.3)	
	≥4,000	13 (12.5)	19 (9.0)	32 (10.1)	
Labor duration (hours)	<24	96 (90.6)	205 (96.7)	301 (94.7)	0.022
	≥24	10 (9.4)	7 (3.3)	17 (5.3)	
Labor monitored with partograph	Yes	81 (76.4)	200 (94.3)	281 (88.4)	0.000
	No	25 (23.6)	12 (5.7)	37 (11.6)	
AMTSL	Yes	86 (81.1)	204 (96.2)	290 (91.2)	0.000
	No	20 (18.9)	8 (3.8)	28 (8.8)	
Third stage prolonged	Yes	25 (23.6)	14 (6.6)	39 (12.3)	0.000
	No	81 (76.4)	198 (93.4)	279 (87.7)	
Perineal tear/lacerations	Yes	7 (6.6)	8 (3.8)	15 (4.7)	0.262
	No	99 (93.4)	204 (96.2)	303 (95.3)	
Safe childbirth checklist	Yes	84 (79.2)	183 (86.3)	267 (84.00)	0.105
	No	22 (20.8)	29 (13.7)	51 (16.0%)	

### 3.5. Risk factors of primary postpartum hemorrhage

To assess the correlation between the occurrence of the dependent variables and the independent factors, we utilized binary logistic regression. The model showed a statistically significant association between primary PPH and factors such as lack of ANC, pregnancy complications, place of birth, way of admission, induced labor, prolonged labor (≥24 h), cesarean delivery, mothers without documented partograph, abnormalities in the third stage of labor and absence of AMTSL.

Variables that were found to be associated with the dependent variable in the bivariate analysis (*P* ≤ 0.05) were taken to the multivariable analysis. After adjusting for possible confounding factors in the multivariable logistic regression, third stage abnormality [AOR = 5.86, 95%, CI (2.55–13.43), *P* = 000], Cesarean section [AOR = 5.61, 95%, CI (2.79–11.30), *P* = 0.000], non-use of AMTSL [AOR = 3.88, 95%, CI (1.29–1.60), *P* = 0.015], non-utilization of partograph [AOR = 3.82, 95%, CI (1.31–11.09), *P* = 0.014], lack of ANC [AOR = 2.76, 95%, CI (1.13–6.75), *P* = 0.026] and complications during pregnancy [AOR = 2.79, 95%, CI (1.34–5.83), *P* = 0.006] were found to be independent predictors of primary PPH (Table 5).

**Table 5:** Multivariate analysis of the association between independent factors and primary postpartum hemorrhage among postnatal mothers in a public hospital in southern Tigray, Ethiopia, 2019.

Variable	Cases	Controls	COR (95% CI)	AOR (95% CI)	*P*-value
**ANC visit**					
Yes	86 (81.1)	199 (93.9)	R	R	
No	20 (18.9)	13 (6.1)	3.56 (1.69–7.48)	2.76 (1.13–6.75)	0.026
**Complications**					
Yes	26 (24.5)	29 (13.7)	2.05 (1.13–3.70)	2.79 (1.34–5.83)	0.006
No	80 (75.5)	183 (86.3)	R	R	
**Place of birth**					
Institutional	97 (91.5)	206 (97.2)	R	R	
Non-institutional	9 (8.5)	6 (2.8)	3.18 (1.10–9.20)	2.26 (0.92–5.56)	0.482
**Way of admission**					
Non-referral	84 (79.2)	186 (87.7)	R	R	
Referral	22 (20.8	26 (12.3)	1.87 (1.00–3.49)	1.08 (0.42–2.22)	0.986
**Way of labor initiated**					
Spontaneously	91 (85.8)	197 (92.9)	R	R	
Induced	15 (14.2)	15 (7.1)	2.50 (1.19–5.24)	2.37 (0.97–5.79)	0.074
**Mode of delivery**					
SVD	67 (63.2)	178 (84.0)	R	R	
C/S	34 (32.1)	25 (11.8)	3.61 (2.00–6.50)	5.61 (2.79–11.30)	0.000
Assisted vaginal delivery	5 (4.7)	9 (4.2)	1.47 (0.47–4.56)	1.81 (0.49–6.60)	
**Labor duration (hours)**					
<24	96 (90.6)	205 (96.7)	R	R	
≥24	10 (9.4)	7 (3.3)	3.05 (1.12–8.25)	2.47 (0.77–7.90)	0.126
**Labor monitored**					
Yes	81 (76.4)	200 (94.3)	R	R	
No	25 (23.6)	12 (5.7)	5.14 (2.46–10.72)	3.82 (1.31–11.09)	0.014
**Abnormal third stage**					
Yes	25 (23.6)	14 (6.6)	R	R	
No	81 (76.4)	198 (93.4)	4.36 (2.16–8.82)	5.86 (2.55–13.43)	0.000
**AMTSL**					
Yes	86 (81.1)	204 (96.2)	R		
No	20 (18.9)	8 (3.8)	5.93 (2.51–13.98)	3.88 (1.29–11.60)	0.015

R, reference.

## 4. Discussion

This study was intended to identify the relative contributions of different factors to the development of primary PPH. Accordingly, the abnormal third stage of labor, non-use of essential maternal health services (ANC, AMTSL and partograph for labor monitoring), cesarean delivery and complications during pregnancy were found to be risk factors of primary PPH.

An abnormal third stage of labor was found to be the strongest independent risk factor for primary PPH in this study; Odds of 3rd stage abnormality were six times higher among cases as compared to controls. This is in line with a study done in Australia and Egypt (35, 36). It is because the longer the duration of the third stage, the greater the blood loss (a logistic regression result revealed that for every five-minute duration before delivery of the placenta, bleeding increased by 40 ml) (34).

Lack of ANC was also found to be an important predictor of primary PPH; the odds of non-utilization of the ANC were higher among cases compared to controls. This finding is in line with a prior study conducted at the Dessie referral hospital (7). This may be due to the fact that mothers who don't have ANC Visits are more likely to experience undiagnosed complications and non-institutional delivery, both of which increase the risk of primary PPH development. Similarly, this study demonstrated that non-use of AMTSL was a predictor of primary PPH, which is consistent with findings from other studies (17, 28, 34, 36). The routine use of AMTSL is recommended by the World Health Organization (WHO) as it decreases the incidence of PPH, the amount of blood loss and the need for blood transfusions (40).

Likewise, mothers who have PIH were at higher risk of developing primary PPH as compared with Mothers without complications, which is congruent with research findings from Parakou (19), Norway (22), and Japan (43). A potential mechanism might be due to the possibility that women with PIH might have hematological abnormalities like abnormal plasma clotting factors, inhibition of platelet activity results in prolonged bleeding time and use of magnesium sulfate facilitates blood loss *via* vasodilatation, a tocolytics effect predisposes to uterine atony, a major cause of PPH (43).

The findings of this study also revealed that C/S increased the risk of Primary PPH by five times. This finding is comparable with a previous study carried out in Ethiopia, Uganda, and Mexico (7, 29, 43) which found that cesarean delivery was a risk factor for primary PPH across all deliveries. The fivefold risk associated with C/S may be because it is frequently the last option when other delivery methods are not possible (such as placenta previa, dehiscence of previous CS scar, uterine rupture) and all of these factors increase the risk of massive blood loss. Women who have C/S are also at risk for several intraoperative issues, such as bleeding and damage to nearby organs (44).

Utilization of a partograph was another important predictor of primary PPH; mothers with primary PPH were four times more likely to experience unmonitored labor with partograph than mothers without primary PPH. This finding is in line with the prior study conducted in Parakou (19). The possible reason for the association here might be partograph is not given enough attention by medical personnel, labor may not be monitored properly and if labor is not monitored effectively, it may become prolonged or unneeded interventions, like labor augmentation and cesarean sections, may be carried out (41).

In some studies, Extreme lower or higher ages in pregnancy (age <20 and >35 years) and grand multiparity has been suggested as a risk factor for PPH (9); however, this study found no association. The disparity could be explained by the fact that the majority of the respondents in this study were between the ages of 20 and 34 years and had a parity of 2–4.

In prior studies illiteracy or primary level schooling and from a rural vicinity of residence, had been reported to be a risk factor for PPH (19), but the current study found no evidence of this. The possible reason might be the continuous health education program offered by health extension employees in the network and household ranges may have contributed to having a similar level of awareness of the issue, which in turn could make the difference undetectable.

In summary, this study generally indicated that primary PPH was linked to the underutilization of essential maternal health care services. In order to further decrease maternal mortality in Ethiopia, those services need to be reinforced. Stakeholders and decision-makers should place a specific emphasis on implementing those life-saving measures.

### 4.1. Limitations and strengths of the study

This study's use of data from several sources (interview and chart review), which can add more precise information and improve data quality, is one of its strengths. Another issue is that case-control studies are preferable to cross-sectional studies for identifying multiple risk factors of a single outcome variable. Being institutional-based and limited generalizability are some limitations.

## 5. Conclusion

Abnormality in the third stage of labor, cesarean route of delivery, non-utilization of ANC, AMTSL, partograph for labor monitoring as well as complications during pregnancy were found to be risk factors of primary postpartum hemorrhage. Therefore, special emphasis should be placed on a strategy towards strengthening the universal use of active management of the third stage of labor for all labors and improving antenatal care coverage, and detecting and handling complications promptly to avoid or decrease the occurrence of primary postpartum hemorrhage and its consequences. Physicians should try options for simple conservative and less invasive routes of delivery initially, and the decision must be with a clear indication to decrease complications associated with cesarean delivery.

## Data Availability

The raw data supporting the conclusions of this article will be made available by the authors, without undue reservation.
